# Differential Associations of Dietary Inflammatory Index and Dietary Antioxidant Indices With Mortality Risk From MASLD/MetALD: Results From a Large Population‐Based Study

**DOI:** 10.1002/fsn3.72429

**Published:** 2026-10-06

**Authors:** Haixin Lyu, Haiyan Chen, Zegeng Zhan, Yangyang Qin, Lifang Mao, Meiyan Guo, Yaxian Mo, Tianran Shen

**Affiliations:** ^1^ Department of Nutrition and Food Hygiene, School of Public Health Guangdong Pharmaceutical University Guangzhou Guangdong China; ^2^ Shenzhen Bao'an Medical College of Guangdong Pharmaceutical University Shenzhen Guangdong China; ^3^ Shenzhen Bao'an District Songgang People's Hospital Shenzhen Guangdong China

**Keywords:** antioxidant, diet, inflammatory, MASLD, MetALD, mortality

## Abstract

Metabolic dysfunction‐associated steatotic liver disease (MASLD) and its subtype, metabolic dysfunction and alcohol‐associated liver disease (MetALD), represent escalating global health challenges. Despite the central role of dietary intervention in current guidelines, evidence remains limited regarding the synergistic impact of anti‐inflammatory and antioxidant diets on the survival benefits of steatotic liver disease (SLD) populations, particularly those with moderate alcohol consumption. This study aims to elucidate the associations between dietary inflammatory/antioxidant quality and mortality across SLD subtypes, and provide insights into dietary recommendations for this patient group. Data from 3048 individuals (2146 MASLD, 902 MetALD) who participated in the 2005–2018 National Health and Nutrition Examination Survey were analyzed. Weighted Cox regression analysis was used to estimate the hazard ratios (HRs) and 95% confidence intervals (CIs) for mortality risk assessment. The mediation effects of inflammation‐related indicators were explored by mediation analysis. During the follow‐up period, a total of 306 deaths were documented. In MASLD patients, higher DII (T3) correlated with increased all‐cause mortality (HR = 1.97, 95% CI: 1.12–3.47, *p* = 0.019). Both CDAI and DAQS showed negative associations, with HRs of 0.50 (0.28–0.89) and 0.47 (0.24–0.93) for T3, respectively (both *p* < 0.05). In MetALD patients, higher DII was associated with increased mortality risk (T2 HR = 4.95, T3 HR = 3.91, both *p* < 0.05). Joint analysis suggested that higher dietary antioxidant quality was associated with lower mortality risks across SLD subtypes compared with the MASLD group with low dietary antioxidant quality. Mediation analysis suggested that inflammation‐related markers (platelets and PHR) may be involved in the associations between dietary indices and mortality in MASLD patients, whereas no statistically significant mediation effects were observed in the MetALD subgroup. Our findings show the key role of dietary quality in reducing mortality among patients with SLD, suggesting that anti‐inflammatory and antioxidant‐rich diets can improve long‐term survival across the SLD spectrum. The findings of this study are expected to provide a new perspective on dietary interventions as a complement to current international guidelines for MASLD/MetALD.

## Graphical abstract

This study investigated the associations of dietary inflammatory and antioxidant quality with mortality risk among individuals with MASLD and MetALD using data from the 2005 to 2018 NHANES. Higher Dietary Inflammatory Index (DII) was associated with increased mortality, whereas higher Composite Dietary Antioxidant Index (CDAI) and Dietary Antioxidant Quality Score (DAQS) were associated with lower mortality risk. These findings highlight the potential importance of anti‐inflammatory and antioxidant‐rich diets in dietary strategies for SLD management.

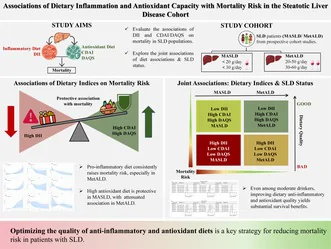

**Abbreviations**
ALT: Alanine aminotransferase
AST: Aspartate aminotransferase
BMI: Body mass index
CDAI: Composite Dietary Antioxidant Index
DAQS: Dietary Antioxidant Quality Score
DII: Dietary Inflammatory Index
FIB‐4: Fibrosis‐4
FLI: Fatty liver index
HDL‐C: High‐density lipoprotein cholesterol
HLP: Hyperlipidaemia
MAFLD: Metabolic dysfunction‐associated fatty liver disease
MASLD: Metabolic dysfunction‐associated steatotic liver disease
MetALD: Metabolic and alcohol‐associated liver disease
NAFLD: Nonalcoholic fatty liver
NHANES: National Health and Nutrition Examination Survey
NLR: Neutrophil count to lymphocyte count ratio
PHR: Platelet count to high‐density lipoprotein cholesterol ratio
PLR: Platelet count to lymphocyte count ratio
RCS: Restricted cubic spline regression
SII: Platelet count multiplied by the neutrophil count to lymphocyte count ratio
SLD: Steatotic liver disease
UA: Uric acid
UHR: Uric acid to high‐density lipoprotein cholesterol ratio

## 1 Introduction

Metabolic dysfunction‐associated steatotic liver disease (MASLD), formerly known as non‐alcoholic fatty liver disease (NAFLD), is a major and growing global health challenge. It places a heavy public health burden on most parts of the world (Younossi et al. 2018). The consensus statement released by EASL (Rinella, Lazarus, et al. 2023), proposed the new term “MASLD”, which definition is more rigorous and multi‐factor limited, aims to more accurately reflect the underlying pathophysiology and metabolic factors driving the disease (Rinella and Sookoian 2024). Meanwhile, a new category named “Metabolic dysfunction and alcohol‐associated liver disease” (MetALD) has been added to describe patients with MASLD who consume a moderate amount of alcohol. Progression to steatohepatitis followed by development of cirrhosis and hepatocellular carcinoma will lead to irreversible deterioration if no intervention is implemented during the MASLD stage (Sheka et al. 2020). Interventions for MetALD patients who have moderate alcohol intake hold significant clinical importance, as this condition is associated with increased mortality risk and more severe hepatic outcomes compared to MASLD alone (Ayares et al. 2025). Currently, although the FDA has approved Resmetirom as the first drug treating Metabolic Dysfunction‐Associated Steatohepatitis (MASH) (Harrison et al. 2024), effective and precise treatment approaches for MASLD or MetALD remain limited (Cusi et al. 2022; Cariou 2022). Therefore, current preventive recommendations focus more on dietary improvements. This necessitates a mechanistic investigation into how dietary interventions can prevent the progression of MASLD or MetALD to more severe steatohepatitis (MASH).

MASLD/MetALD are both characterized by an imbalance of lipid metabolism, chronic inflammation (Yang et al. 2026) and oxidative stress (Wang et al. 2025). As a modifiable factor, dietary nutrients may play an important role in regulating oxidative stress and inflammation in humans (Li et al. 2024). With increasing emphasis on lifestyle factors, research on inflammatory and antioxidant diets has grown substantially. The Dietary Inflammatory Index (DII) was developed to evaluate dietary patterns by integrating evidence from various populations through systematic research methods and nutritional assessment approaches (Shivappa et al. 2014). Higher DII scores indicate greater pro‐inflammatory effects of the diet, while lower scores reflect stronger anti‐inflammatory properties (Li et al. 2023). The Composite Dietary Antioxidant Index (CDAI) and Dietary Antioxidant Quality Score (DAQS), which include vitamins A/C/E, zinc, selenium, and carotenoids, quantitatively evaluate total dietary antioxidant consumption patterns at the individual level. Substantial evidence has shown an association between a high DII and the development of MASLD (Yang et al. 2025). Recently, Tao et al. (2025) demonstrated that an elevated DII is associated with increased mortality risk in adults with MASLD. However, it remains unknown whether this association extends to other types within the steatotic liver disease (SLD) spectrum, particularly MetALD, which characterizes individuals with moderate alcohol consumption. Furthermore, while some studies emphasize a significant inverse relationship between the CDAI and MASLD prevalence (He et al. 2025), other dimensions of antioxidant dietary quality and total antioxidant capacity have not been systematically evaluated regarding their long‐term prognostic impact. To date, limited prospective evidence has evaluated the association between antioxidant dietary patterns and mortality outcomes among individuals with steatotic liver disease (SLD) according to the newly proposed MASLD/MetALD classification framework. In addition, whether inflammation‐related markers mediate the associations between dietary antioxidant capacity and mortality risk in SLD populations remains unclear.

Despite evidence that indices such as the DII and dietary antioxidant metrics (CDAI and DAQS) are closely associated with metabolic diseases, there remains a lack of systematic analysis on how dietary inflammatory and antioxidant quality jointly influence the long‐term prognosis of MASLD and its MetALD under the context of the redefinition of SLD. In particular, the potential role of dietary quality in modifying prognosis among individuals with alcohol exposure‐related SLD remains insufficiently understood. The harmful effects of long‐term heavy drinking on the liver have been substantiated by evidence (Devarbhavi et al. 2023). Nevertheless, the health effects of moderate alcohol consumption remain controversial. Some epidemiological studies suggest that, compared to not drinking at all, having one to two drinks per day is associated with lower risks of death, cardiovascular disease (Mostofsky et al. 2016), and Type 2 diabetes (T2DM) (Knott et al. 2015). Additionally, some studies have suggested that moderate wine consumption within the context of high‐quality dietary patterns, such as the Mediterranean diet, may not increase metabolic risk among individuals with T2DM (Ma et al. 2022). However, these observations remain subject to debate, as even relatively low levels of alcohol intake may be associated with adverse health outcomes (Wood et al. 2018). Therefore, among individuals with MetALD characterized by both metabolic dysfunction and moderate alcohol exposure, whether adherence to low‐inflammatory and high‐antioxidant dietary patterns is associated with improved survival outcomes remains unclear.

Based on the above background, this study aims to: First, clarify the independent long‐term prognostic impact of pro‐inflammatory diets and antioxidant status on patients with different subtypes of SLD; second, explore, through joint analysis, the benefits of dietary quality and SLD subtype in reducing mortality risk; and finally, investigate differential mechanistic pathways among subgroups using mediation effect models. The findings of this study are expected to provide a new perspective on dietary interventions as a complement to current international guidelines.

## 2 Materials and Methods

### 2.1 Study Design and Population

The National Health and Nutrition Examination Survey (NHANES), administered by CDC's National Center for Health Statistics, assesses the health and nutrition status of the US population through interviews, physical exams, and lab tests. It gathers data on demographics, dietary intake, medical history, and biomarkers. All research data are publicly available through open‐access platforms (website: https://www.cdc.gov/nchs/nhanes/index.htm). The NHANES adhered to strict ethical standards including institutional review board (National Center for Health Statistics Research Ethics Review Board) approval and documented informed consent from all enrolled subjects.

Data from NHANES 2005–2018 about 7 cycles were selected for study. The study ultimately enrolled 3048 subjects from an initial population of 70,190. Exclusion criteria included: individuals under the age of 20 years old; individuals with missing variables for calculating FLI; pregnant individuals; individuals with missing dietary data; individuals without SLD; female individuals whose alcohol intake > 50 g/day or male individuals whose alcohol intake > 60 g/day; individuals with viral hepatitis. According to the latest consensus diagnostic criteria from the EASL‐AASLD‐ALEH consensus statement in 2023 (European Association for the Study of the Liver [EASL] et al. 2024), 2146 people were diagnosed with MASLD, and 902 people were diagnosed with MetALD. The details are presented in Figure 1.

**FIGURE 1:**
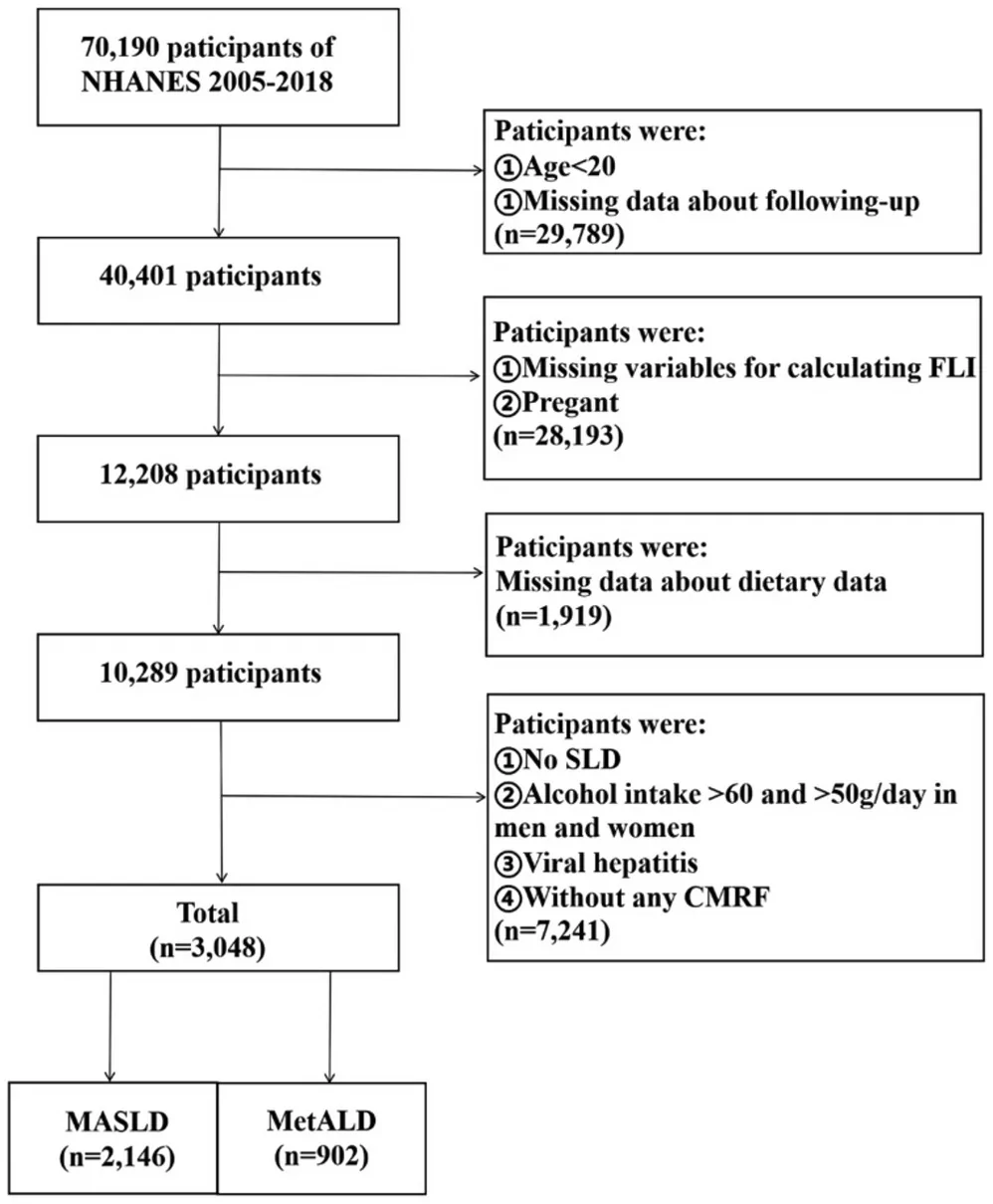
Flowchart of participants' selection.

### 2.2 Definition of MASLD/MetALD

Hepatic steatosis was assessed using the FLI, as specific imaging or histological data related to fatty liver were not available in the NHANES 2005–2018. The FLI is based on BMI, waist circumference, triglycerides, and gamma‐glutamyltransferase (GGT) and this index shows a good diagnostic performance with an area under the receiver operator curve (AUROC) of 0.85 (Bedogni et al. 2006). Using An FLI ≥ 30 indicated hepatic steatosis. Although the traditional diagnostic criterion for hepatic steatosis has been FLI ≥ 60, an FLI < 30 demonstrates extremely high sensitivity, enabling the exclusion of this condition. In our study, we deliberately adopted a lower threshold (≥ 30) to maximize detection sensitivity, thereby allowing for the identification of early‐stage hepatic fat accumulation and encompassing individuals within the metabolic high‐risk range (Castellana et al. 2021). Cardio‐metabolic risk factors (CMRF) criteria included: (1) body mass index ≥ 25 kg/m2 or waist circumference > 94/80 cm in men and women, (2) fasting glucose ≥ 100 mg/dL or HbA1c ≥ 5.7% or Type 2 diabetes or treatment for Type 2 diabetes, (3) blood pressure ≥ 130/85 mmHg or antihypertensive treatment, (4) plasma triglycerides ≥ 150 mg/dL or lipid‐lowering treatment, and (5) plasma HDL cholesterol ≤ 40 mg/dL in men and ≤ 50 mg/dL in women or lipid‐lowering treatment (Kendel Jovanovic et al. 2020).

After excluding participants who consumed more than 60 g/day of alcohol for males or more than 50 g/day for females, those with viral hepatitis, those without steatotic liver disease, and those without any CMRF, a population characterized by steatotic liver disease was obtained (total). Subsequently, subtypes of MASLD and MetALD were identified within this cohort.

MASLD is defined as the presence of fatty liver detected by FLI and matching at least one CMRF criterion, with low alcohol consumption—> 30 g/day for men and > 20 g/day for women.

MetALD is defined as hepatic steatosis detected by FLI that matches at least one of the following five specific criteria, accompanied by moderate alcohol consumption: 30–60 g/day for males and 20–50 g/day for females.

In addition, for ease of subsequent description, the alcohol consumption criterion for MASLD in this study was termed low consumption, whereas the alcohol consumption criterion for MetALD, which lies between that of MASLD and ALD, was termed moderate consumption.

### 2.3 Mortality and Follow‐Up

The outcome was all‐cause mortality and CVD mortality in the MASLD/MetALD population. The causes of death included CVD, respiratory disease, malignancy, diabetes, Alzheimer's disease, nephrology‐related diseases, and accidental death. Deaths from CVD were identified using the International Statistical Classification of Diseases, 10th Revision (ICD‐10) codes I00–I09, I11, I13, I20–I51, and I60–I69. The follow‐up time of the study was calculated from the NHANES 2005–2018 examination date until the last known date alive or censored through 31 December 2019.

### 2.4 Dietary Assessment

Dietary intake data were collected through 24‐h dietary recall interviews conducted by trained interviewers via face‐to‐face interviews at mobile examination centers (MECs). Participants were asked to recall all foods and beverages (excluding plain water) consumed during the 24 h prior to the interview. Dietary antioxidant and total energy intake values were calculated using the USDA Food and Nutrient Database for Dietary Studies (FNDDS) (Ahuja et al. 2013).

### 2.5 Definition of DII

The DII can serve as a simple tool to evaluate the inflammatory potential of daily dietary intake. It was created by Shivappa et al. (2014), which consists of 45 dietary components or nutrients. It can be utilized as a method for dietary quality evaluation to determine whether a certain diet has the potential to cause inflammation. The pro‐inflammatory effect of diet develops with a greater DII score, while its anti‐inflammatory effect improves with a lower DII score. The DII's specifics are available elsewhere (Zhang et al. 2024). Overall, processing raw data, calculating *Z*‐scores, converting the centered percentiles, and multiplying the “overall inflammatory effect score” are the steps in the calculation for a specific food parameter's DII. The sum of the DII for all food components represents the final DII score. The present investigation excluded elements that were a part of the initial DII computation, such as flavan‐3‐ol, flavones, flavonols, flavonones, anthocyanidins, isoflavones, pepper, thyme/oregano, and rosemary, since the NHANES 2005–2018 data did not have them accessible. According to the dietary interview provided by NHANES, DII was calculated using 28 out of 45 food components (Zhao et al. 2023). The majority of the food items that are absent are often consumed in little amounts by this population, and therefore their absence is probably not going to have a big impact on the overall DII ratings. The ultimate result of DII was calculated by two 24‐h dietary recall interview data. In this study, we divided the DII score into three equal parts on average to evaluate low (T1), moderate (T2), and high DII levels (T3).

### 2.6 Definition of CDAI

The CDAI can serve as a simple tool to assess overall dietary antioxidant exposure. A modified version of the CDAI developed by Wright et al. (2004) was used, where higher CDAI scores indicate greater total dietary antioxidant capacity. The first recall was taken in‐person at a mobile testing center, while the second recall was performed 3–10 days later via a telephone consultation. The CDAI of each participant was calculated based on the dietary recall of intake of six dietary antioxidants, vitamin A, vitamin C, vitamin E, zinc, selenium, and carotenoids. The ultimate result of CDAI was calculated by two 24‐h dietary recall interview data. In this study, we divided the CDAI score into three equal parts on average to evaluate low (T1), moderate (T2), and high CDAI levels (T3).

### 2.7 Definition of DAQS

The DAQS can serve as a simple tool to evaluate the quality of daily antioxidant intake. It was assessed based on the six dietary antioxidant nutrients, including vitamin A, vitamin C, vitamin E, zinc, selenium, and carotenoids. Each of those six nutrients/minerals' intake was compared with their respective recommended daily intake (RDI) for US adults released by the Dietary Guidelines for Americans 2015–2020 (Wang et al. 2022). The DAQS of 0 and 1 were defined as intake < 2/3 of RDI and intake ≥ 2/3 of the RDI for each nutrient/mineral, respectively. The DAQS ranged from 0 (poor quality) to 6 (high quality). In our study, DAQS were divided into three groups: 0–2, 3–4, and 5–6. The ultimate result of DAQS was calculated by two 24‐h dietary recall interview data.

### 2.8 Inflammation‐Related Indicators

Data for indicators were obtained from the laboratory data section of the NHANES database. Following standardized procedures, skilled healthcare professionals at the mobile examination center employed rigorous techniques and state‐of‐the‐art equipment to measure neutrophil count, lymphocyte count, and platelet count. The UHR of each participant was determined by the uric acid to high‐density lipoprotein cholesterol ratio. The NLR was determined by the neutrophil count to lymphocyte count ratio. The PLR level was determined by the platelet count to lymphocyte count ratio. The SII level was determined by the platelet count multiplied by the neutrophil count to lymphocyte count ratio. The PHR level was determined by the platelet count to high‐density lipoprotein cholesterol ratio.

### 2.9 Covariates and Stratification Variables

The covariates associated with individual characteristics included age, sex, ethnicity, income, body mass index (BMI), smoking status, sitting time, total energy intake, dietary supplement use, cancer, diabetes, hyperlipidaemia (HLP), alanine aminotransferase (ALT), aspartate aminotransferase (AST), uric acid (UA). All detailed measurement procedures are available at the NHANES official website. Diabetes was diagnosed based on self‐reports, a HbA1c > 6.5%, fasting blood glucose > 7.0 mmoL/L, or the use of anti‐diabetic medication. Cancer and HLP status were defined based on participants' self‐reported physician diagnosis of cancer and HLP from the NHANES questionnaire. This choice of covariates is made to mitigate the recall bias associated with questionnaire‐based data collection. Participants who smoked at least 100 cigarettes in their life were defined as smokers, regardless of whether they had quit smoking at the time of the interview. A BMI score > 18.5 was categorized as underweight, 18.5–25 as normal weight, 25–30 as overweight, and above 30 as obesity.

In the subgroup analysis, the Fibrosis‐4 (FIB‐4) index was included as an additional variable. The FIB‐4 index incorporates parameters such as age, AST, ALT, and platelet count (with an area under the receiver operating characteristic curve (AUROC) of 0.80). A FIB‐4 value > 1.3 indicates mild fibrosis, 1.3–2.67 indicates moderate fibrosis, and above 2.67 can be used to diagnose significant fibrosis (European Association for the Study of the Liver [EASL] et al. 2024).

### 2.10 Statistical Analysis

All statistical analyses were performed by SAS 9.4 (SAS Institute Inc., Cary, NC, USA) and R software (version 4.4.2, Vienna, Austria: R Foundation for Statistical Computing, 2016), with bilateral *p* values < 0.05 defined as statistically significant. Statistical analysis followed the analytic and reporting guidelines for NHANES, using the sampling weights and the complex survey design to represent a nationally representative level of the US population. Participants were initially screened based on the study's inclusion and exclusion criteria. Individuals with missing data for dietary indices and key variables required for the identification of MASLD/MetALD were excluded. Among the remaining participants, the proportion of missing values across all covariates was minimal (> 1% for each variable) or non‐existent. These missing values were addressed using mean imputation for continuous covariates and mode imputation for categorical covariates. Continuous variables were expressed as weighted means (SD), and categorical variables were expressed as numbers (percentages). Between‐group comparisons were performed using a one‐way ANOVA for continuous variables and chi‐square tests for categorical variables. Missing data were supplemented using the mean imputation method.

Kaplan–Meier method was used to plot the survival curves associated with different classification methods for DII, CDAI, and DAQS. After that, the log‐rank test was used to compare differences between survival curves. DII, CDAI, and DAQS were converted into categorical variables based on tertile divisions.

Multivariable weighted Cox regression analysis was used to estimate the hazard ratios (HRs) and 95% confidence intervals (CIs) for the associations between the DII, CDAI, DAQS, and all‐cause mortality or CVD mortality in patients with MASLD/MetALD. Three models were conducted: Model 1 adjusted for age, sex, ethnicity, and income; Model 2 adjusted for age, sex, ethnicity, income, body mass index, smoking status, and sedentary time; Model 3 adjusted for age, sex, ethnicity, income, body mass index, smoking status, sedentary time, total energy intake, use of dietary supplements, cancer, diabetes, ALT, AST, UA, and HLP.

Restricted cubic spline regression (RCS) with 3 knots was used to analyze the nonlinear relationships between the DII and mortality, between the CDAI and mortality, as well as between the DAQS and mortality.

To examine the joint association between dietary indicators and SLD subtype on all‐cause and CVD mortality risk, we incorporated a multiplicative term between dietary indicators and SLD status (MASLD, MetALD) into the Cox regression model to explore potential interactions on a multiplicative scale. Participants were categorized into six patterns based on the inflammatory or antioxidant properties of their diet and the SLD status. Pattern 1: patients with MASLD who exhibited a pro‐inflammatory or low‐antioxidant dietary pattern. Pattern 2: patients with MASLD who exhibited a moderately anti‐inflammatory or moderately antioxidant dietary pattern. Pattern 3: patients with MASLD who exhibited an anti‐inflammatory or high‐antioxidant dietary pattern. Pattern 4: patients with MetALD who exhibited a pro‐inflammatory or low‐antioxidant dietary pattern. Pattern 5: patients with MetALD who exhibited a moderately anti‐inflammatory or moderately antioxidant dietary pattern. Pattern 6: patients with MetALD who exhibited an anti‐inflammatory or high‐antioxidant dietary pattern. Weighted Cox proportional hazards regression models were employed to calculate hazard ratios (HRs) and 95% confidence intervals (CIs) for all‐cause mortality risk across the six patterns. Finally, forest plots were generated using the “Forest Plot Generation” module of the Mengteyun Statistics Platform (https://mengte.pro/forest_plot).

In addition, we applied a mediation model calculated based on the bootstrap method to investigate the direct effects of DII, CDAI, and DAQS on the all‐cause death risk in the MASLD/MetALD population and the indirect effects mediated by inflammation‐related indicators. Subgroup analyses were conducted by stratifying according to all covariates to explore whether the potential impacts of DII, CDAI, and DAQS on mortality in the entire cohort were homogeneous across subgroups.

Finally, sensitivity analyses were performed by adjusting for hypertension or FIB‐4 to address the potential impacts of hypertension and FIB‐4 on the results.

## 3 Results

### 3.1 Baseline Characteristics

This study included a total of 3048 participants, comprising 2146 individuals diagnosed with MASLD and 902 with MetALD. The mean age was 51.56 ± 0.60 years in the MASLD group, 48.87 ± 1.14 years in the MetALD group, and 50.06 ± 0.52 years in the total population. Regarding gender distribution, females accounted for 57.36% in the MASLD group, while males constituted 56.19% in the MetALD group. Regarding metabolic and liver‐related biomarkers, several significant differences were observed between the two groups. UA levels were significantly higher in the MetALD group (6.11 mg/dL) than in the MASLD group (5.35 mg/dL, *p* < 0.001). Similarly, liver enzyme levels were elevated in MetALD, with higher AST (25.28 U/L, *p* = 0.010) and ALT (25.03 U/L, *p* = 0.003). In terms of hematological inflammatory markers, platelet counts were significantly lower in MetALD (224.69 × 10^3^ cells/μL) compared with MASLD (241.08 × 10^3^ cells/μL, *p* < 0.001). For composite inflammatory indices, the UHR was significantly higher in MetALD (0.113 vs. 0.100, *p* < 0.001). Conversely, the PLR and PHR were significantly lower in MetALD than in MASLD (PLR: 124.70 vs. 133.53, *p* = 0.004; PHR: 4.16 vs. 4.45, *p* = 0.014). Dietary indicators also differed significantly. The DII was lower in the MetALD group compared with MASLD (0.85 vs. 1.55, *p* < 0.001). In contrast, the CDAI and the DAQS were significantly higher in MetALD (CDAI: 4.94 vs. 1.75; DAQS: 3.64 vs. 3.26; both *p* < 0.001). General characteristics of the study population are presented in Table 1.

**TABLE 1:** Baseline characteristics in the National Health and Nutrition Examination Survey (2005–2018).

	Total	MASLD	MetALD	*p*
	*N* = 3048	*N* = 2146	*N* = 902	
Age, year	50.06 (0.52)	51.56 (0.60)	48.87 (1.14)	< 0.001
Sex, %				
Male	9,311,486 (47.31)	5,500,006 (42.64)	3,811,479 (56.19)	< 0.001
Female	10,372,192 (52.69)	7,399,993 (57.36)	2,972,199 (43.81)	
Ethnicity, %				
Mexican American	1,245,896 (6.33)	807,089 (6.26)	438,807 (6.47)	< 0.001
Other Hispanic	835,983 (4.25)	530,398 (4.11)	305,585 (4.50)	
Non‐Hispanic White	12,023,799 (61.09)	7,545,650 (58.49)	4,478,149 (66.01)	
Non‐Hispanic Black	4,507,978 (22.90)	3,261,328 (25.28)	1,246,650 (18.38)	
Non‐Hispanic Asian	316,247 (1.61)	236,642 (1.83)	79,605 (1.17)	
Other race	753,776 (3.83)	518,893 (4.02)	234,882 (3.46)	
Income, %				
$20,000 and over	19,567,660 (99.41)	12,811,194 (99.31)	6,756,465 (99.60)	0.831
Under $20,000	116,019 (0.59)	88,806 (0.69)	27,213 (0.40)	
Smoking status, %				
Non‐smoker	12,381,812 (62.90)	8,324,585 (64.53)	4,057,227 (59.81)	0.046
Smoker	7,301,866 (37.10)	4,575,415 (35.47)	2,726,451 (40.19)	
BMI, kg/m^2^	31.63 (0.17)	31.94 (0.23)	30.20 (0.32)	< 0.001
Sedentary time, %				
< 4 h	4,437,602 (22.54)	3,109,305 (24.10)	1,328,296 (19.58)	0.028
4–6 h	5,037,462 (25.59)	3,445,677 (26.71)	1,591,785 (23.47)	
6–8 h	3,137,328 (15.94)	2,095,635 (16.25)	1,041,692 (15.36)	
> 8 h	7,071,287 (35.92)	4,249,382 (32.94)	2,821,905 (41.60)	
Total energy intake, kcal	2005.31 (18.98)	1935.67 (24.63)	2385.01 (40.08)	< 0.001
Dietary supplements, %				
Yes	8,997,643 (45.71)	5,856,562 (45.40)	3,141,081 (46.30)	0.244
No	10,686,035 (54.29)	7,043,438 (54.60)	3,642,597 (53.70)	
Cancer, %				
Yes	1,870,773 (9.50)	1,328,498 (10.30)	542,275 (7.99)	0.795
No	17,812,905 (90.50)	11,571,502 (89.70)	6,241,403 (92.01)	
Diabetes, %				
Yes	4,466,941 (22.69)	2,770,227 (21.47)	1,696,715 (25.01)	0.902
No	15,216,737 (77.31)	10,129,773 (78.53)	5,086,963 (74.99)	
FIB‐4, %				
< 1.3	13,189,829 (67.01)	8,238,183 (63.86)	4,951,646 (72.99)	0.276
1.3–2.67	5,594,922 (28.42)	3,957,700 (30.68)	1,637,222 (24.13)	
> 2.67	898,927 (4.57)	704,117 (5.46)	194,810 (2.87)	
UA, mg/gL	5.42 (0.04)	5.35 (0.04)	6.11 (0.08)	< 0.001
AST, U/L	24.10 (0.31)	23.48 (0.30)	25.28 (0.66)	0.010
ALT, U/L	23.06 (0.32)	22.02 (0.32)	25.03 (0.74)	0.003
HDL‐C, mg/dL	58.60 (0.55)	57.66 (0.56)	60.39 (1.04)	0.925
Neutrophil, 1000 cells/μL	4.02 (0.04)	4.00 (0.06)	3.99 (0.10)	0.893
Lymphocyte, 1000 cells/μL	2.01 (0.02)	2.01 (0.03)	1.97 (0.04)	0.436
Platelet, 1000 cells/μL	239.53 (1.99)	241.08 (2.48)	224.69 (3.25)	< 0.001
UHR	0.100 (0.001)	0.100 (0.002)	0.113 (0.002)	< 0.001
NLR	2.22 (0.03)	2.24 (0.04)	2.23 (0.08)	0.924
PLR	131.38 (1.64)	133.53 (1.82)	124.70 (2.76)	0.004
SII	530.10 (8.55)	536.73 (10.18)	501.61 (19.45)	0.127
PHR	4.37 (0.06)	4.45 (0.07)	4.16 (0.09)	0.014
DII	1.45 (0.06)	1.55 (0.06)	0.85 (0.12)	< 0.001
CDAI	2.85 (0.47)	1.75 (0.53)	4.94 (0.86)	< 0.001
DAQS	3.33 (0.04)	3.26 (0.05)	3.64 (0.09)	< 0.001

### 3.2 Relationship of Dietary Inflammatory Index and Dietary Antioxidant Indices With All‐Cause Mortality

Prior to Cox regression analysis, Kaplan–Meier survival curves were constructed and the Log‐rank test was performed. Detailed results are presented in Figure S1. Table 2 presents the results of weighted Cox regression analyses with DII, CDAI, and DAQS categorized into tertiles. Among them, 248 MASLD patients experienced all‐cause mortality, while 58 MetALD patients experienced all‐cause mortality. Overall, DII demonstrated predominantly positive associations with all‐cause mortality, whereas CDAI and DAQS exhibited negative correlations. The magnitude and statistical significance of these associations varied across disease subgroups and model adjustments.

**TABLE 2:** Association of dietary indices with all‐cause mortality in adults with Total, MASLD and MetALD.

		T2		T3		*p*‐trend
	T1	HR	*p*	HR	*p*	
Total						
DII						
Model 1	Ref	1.38 (0.85–2.21)	0.188	1.72 (1.11–2.67)	**0.016**	**0.015**
Model 2	Ref	1.45 (0.90–2.33)	0.122	1.80 (1.17–2.78)	**0.009**	**0.008**
Model 3	Ref	1.67 (1.04–2.69)	0.034	2.04 (1.28–3.27)	**0.003**	**0.004**
CDAI						
Model 1	Ref	0.82 (0.57–1.18)	0.277	0.53 (0.34–0.83)	**0.006**	**0.005**
Model 2	Ref	0.83 (0.59–1.19)	0.316	0.54 (0.35–0.83)	**0.006**	**0.004**
Model 3	Ref	0.88 (0.51–1.18)	0.159	0.44 (0.26–0.74)	**0.003**	**0.002**
DAQS						
Model 1	Ref	0.82 (0.59–1.13)	0.223	0.57 (0.36–0.90)	**0.017**	**0.024**
Model 2	Ref	0.82 (0.60–1.12)	0.211	0.58 (0.37–0.91)	**0.019**	**0.018**
Model 3	Ref	0.82 (0.58–1.15)	0.244	0.57 (0.35–0.92)	**0.021**	**0.025**
MASLD						
DII						
Model 1	Ref	1.00 (0.60–1.66)	0.998	1.50 (0.90–2.50)	0.119	0.135
Model 2	Ref	1.06 (0.64–1.78)	0.815	1.60 (0.97–2.63)	0.066	0.075
Model 3	Ref	1.34 (0.80–2.24)	0.257	1.97 (1.12–3.47)	**0.019**	**0.019**
CDAI						
Model 1	Ref	0.88 (0.58–1.33)	0.527	0.58 (0.34–0.97)	**0.038**	**0.031**
Model 2	Ref	0.90 (0.60–1.36)	0.626	0.61 (0.37–1.00)	**0.050**	**0.040**
Model 3	Ref	0.83 (0.53–1.29)	0.397	0.50 (0.28–0.89)	**0.019**	**0.016**
DAQS						
Model 1	Ref	0.73 (0.47–1.12)	0.146	0.52 (0.27–1.01)	0.054	**0.046**
Model 2	Ref	0.72 (0.48–1.08)	0.105	0.50 (0.26–0.97)	**0.039**	**0.031**
Model 3	Ref	0.67 (0.43–1.04)	0.074	0.47 (0.24–0.93)	**0.031**	**0.025**
MetALD						
DII						
Model 1	Ref	3.51 (1.35–9.12)	**0.010**	2.60 (1.13–6.00)	**0.025**	**0.010**
Model 2	Ref	3.33 (1.33–8.37)	**0.011**	2.48 (1.02–6.03)	**0.045**	**0.025**
Model 3	Ref	4.95 (1.87–13.15)	**0.002**	3.91 (1.46–10.49)	**0.007**	**0.005**
CDAI						
Model 1	Ref	0.34 (0.05–2.28)	0.263	0.29 (0.05–1.71)	0.170	0.086
Model 2	Ref	0.30 (0.03–3.48)	0.334	0.26 (0.05–1.39)	0.114	0.087
Model 3	Ref	0.40 (0.11–1.53)	0.180	0.23 (0.05–1.16)	0.074	0.077
DAQS						
Model 1	Ref	1.41 (0.68–2.90)	0.354	0.81 (0.36–1.79)	0.593	0.495
Model 2	Ref	1.50 (0.71–3.14)	0.283	0.88 (0.41–1.88)	0.730	0.642
Model 3	Ref	1.41 (0.61–3.28)	0.423	0.60 (0.26–1.39)	0.229	0.133

*Note:* Model 1, adjusted for age, sex, ethnicity and income. Model 2, model 1 + BMI, smoking status and sedentary time. Model 3, model 2 + total energy intake, dietary supplements use, cancer, diabetes, ALT, AST, UA and HLP. Bold values indicate *p* < 0.05.

In the total population with SLD characteristics, DII was positively associated with all‐cause mortality (T3 vs. T1: HR 2.04; 95% CI 1.28–3.27), whereas CDAI and DAQS showed protective associations (CDAI: HR 0.44; 95% CI 0.26–0.74; DAQS: HR 0.57; 95% CI 0.35–0.92). A similar trend was also observed in the MASLD population. In the MASLD population, DII was also associated with an increased risk of mortality (HR 1.97; 95% CI 1.12–3.47), while CDAI and DAQS were associated with lower mortality risk (CDAI: HR 0.50; 95% CI 0.28–0.89; DAQS: HR 0.47; 95% CI 0.24–0.93). In the MetALD population, DII showed a stronger consistent positive association with all‐cause mortality (HR 3.91; 95% CI 1.46–10.49), whereas CDAI and DAQS showed inverse trends that did not reach statistical significance (CDAI: HR 0.23; 95% CI 0.05–1.16; DAQS: HR 0.60; 95% CI 0.26–1.39).

Figure 2 used the RCS method to show the associations between dietary indices (DII, CDAI, DAQS) and all‐cause mortality in MASLD and MetALD patients. The results showed that pro‐inflammatory diets (high DII) may increase mortality risk, while antioxidant‐rich diets (high CDAI and DAQS) might reduce it.

**FIGURE 2:**
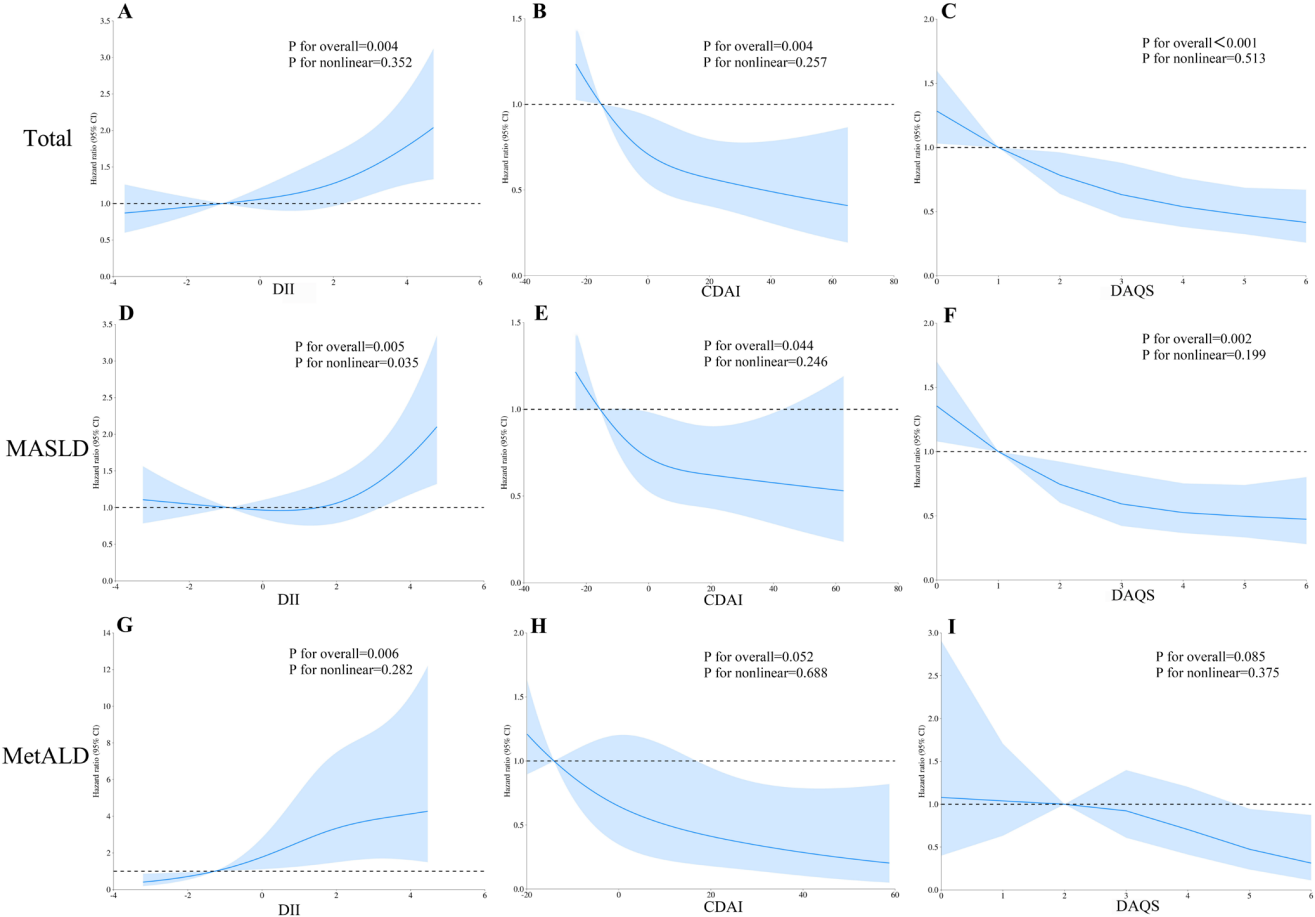
Associations of DII, CDAI and DAQS with all‐cause mortality, evaluated using CPH regression models and RCS after adjusting for age, sex, ethnicity, income, BMI, smoking status, sedentary time, total energy intake, dietary supplements use, self‐reported cancer, diabetes, ALT, AST, UA and HLP. Central estimates are represented by solid blue lines, and blue shaded areas indicate 95% confidence intervals. (A–C) total participants, (D–F) adults with MASLD, (G–I) adults with MetALD.

### 3.3 Relationship of Dietary Inflammatory Index and Dietary Antioxidant Indices With CVD Mortality

Table S1 presents the associations between different dietary indices (DII, CDAI, DAQS) and CVD mortality risk, analyzed separately in the overall population with SLD characteristics (Total), MASLD, and MetALD subgroups. Overall, DII showed a positive trend toward CVD mortality risk, with a particularly significant and stable association in the MetALD subgroup, though most groups failed to reach statistical significance. CDAI exhibited an overall negative association, yet most groups also lacked statistical significance. DAQS demonstrated a relatively consistent protective effect in the Total and MASLD populations, reducing mortality risk in the T3 group (HR 0.42; 95% CI 0.24–0.74), and Model 3 adjusted for covariates in the total cohort showed statistical significance (*p*‐trend = 0.029). A similar trend was observed in the RCS curve, forming an L‐shaped pattern (Figure S2).

### 3.4 Associations of Dietary Indices With Mortality Risk According to SLD Status

Building upon the independent associations observed within each SLD subtype, we conducted a joint analysis across the MASLD and MetALD groups to further explore whether the impact of dietary indices on mortality differed by SLD status, as shown in Figure 3. Using MASLD participants with the poorest diet quality (highest DII or lowest CDAI/DAQS) as the reference, adherence to optimal diets consistently reduced all‐cause mortality across both SLD subtypes. Specifically, the protective association of higher DAQS was observed in both MASLD (HR: 0.380, 95% CI: 0.193–0.747) and MetALD (HR: 0.188, 95% CI: 0.077–0.461), with similar patterns observed for lower inflammatory potential (DII T1) and higher antioxidant intake (CDAI T3). For CVD mortality, higher DAQS was associated with lower CVD mortality risk in MASLD (HR: 0.309, 95% CI: 0.092–1.044); comparatively larger risk reductions were observed among MetALD patients in the highest DAQS and CDAI tertiles (HR: 0.073, 95% CI: 0.015–0.348; HR: 0.154, 95% CI: 0.046–0.514, respectively). Collectively, these findings indicate that optimal dietary patterns attenuate mortality risks across the SLD spectrum, conferring relatively greater benefits to individuals with MetALD.

**FIGURE 3:**
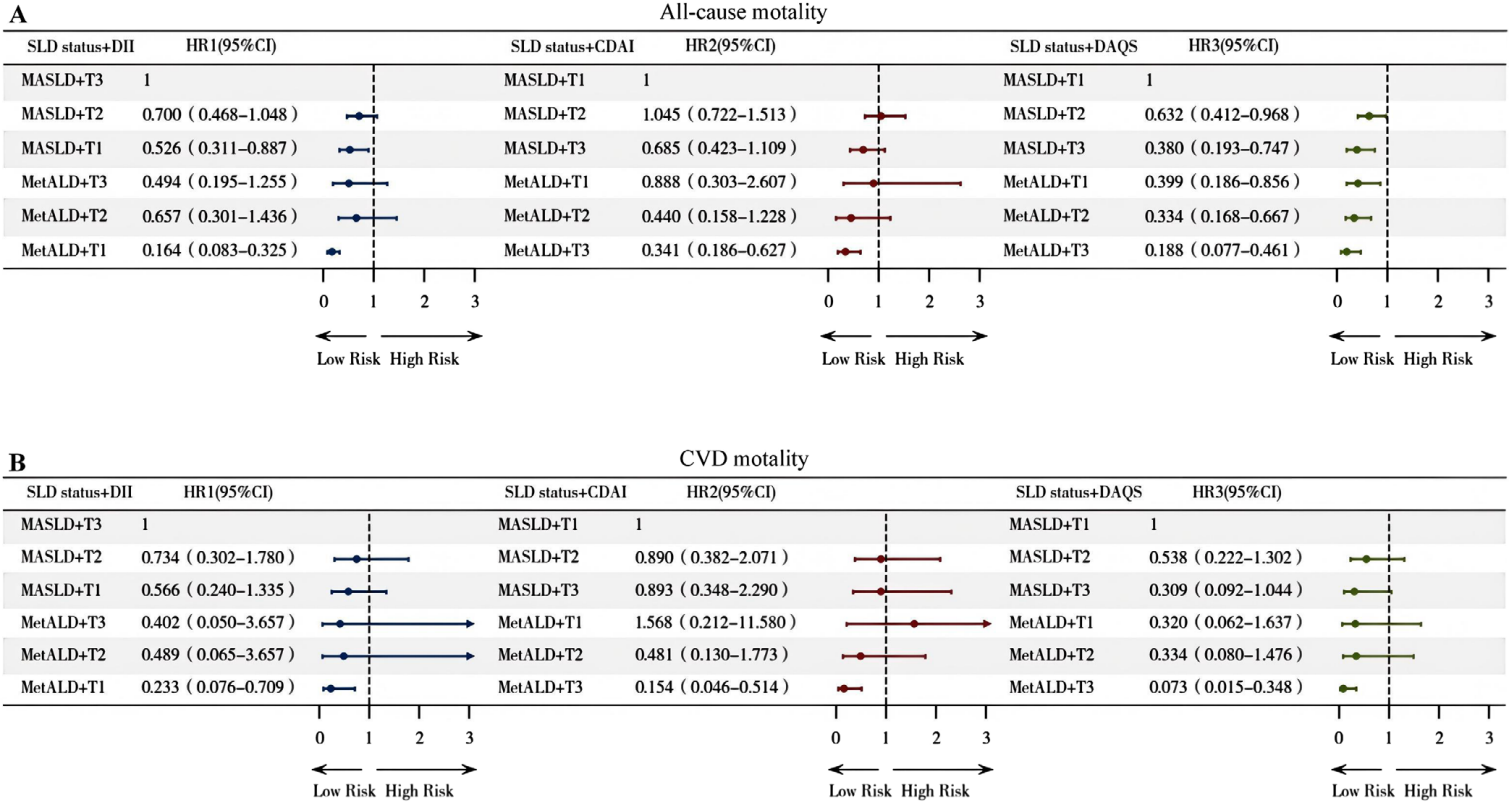
The forest plot of associations between dietary indices and the risk of all‐cause and CVD mortality, stratified by SLD status. Adjusted for age, sex, ethnicity, income, BMI, smoking status, sedentary time, total energy intake, dietary supplements use, self‐reported cancer, diabetes, ALT, AST, UA, and HLP. (A) all‐cause mortality, (B) CVD mortality.

### 3.5 Mediating Role of Inflammation‐Related Indicators

Mediation analyses were further conducted to explore the potential involvement of inflammation‐related indicators in the associations between dietary indices and all‐cause mortality (Table S2; Figure 4).

**FIGURE 4:**
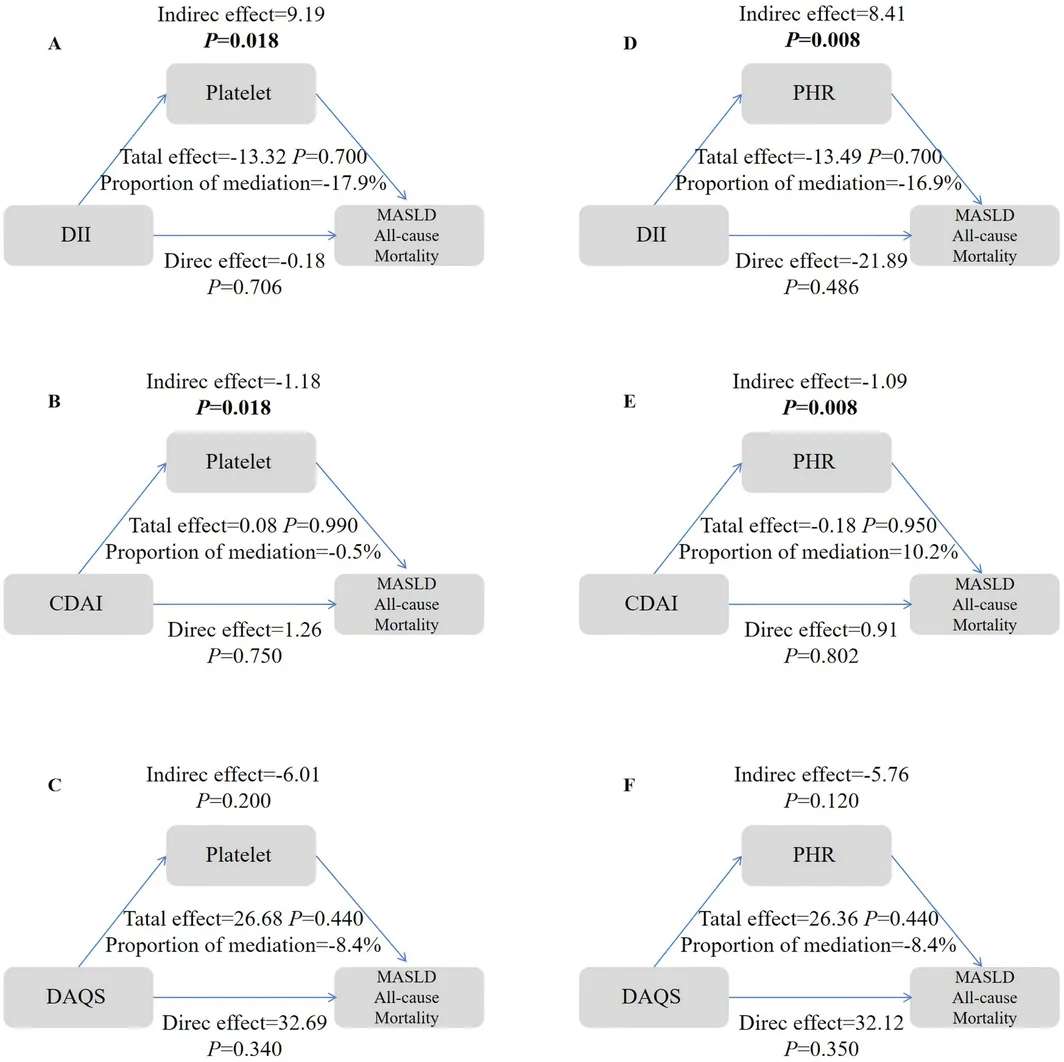
Analysis of the mediation by Platelet (A–C) and PHR (D–F) of the associations of DII, CDAI, and DAQS with all‐cause mortality among adults with MASLD. The 95% CI of these estimates was computed using the bootstrap method (1000 samples). In all mediation analyses, adjustments were made for the following covariates: Adjusted for age, sex, ethnicity, income, BMI, smoking status, sitting time, total energy intake, dietary supplements use, self‐reported cancer, diabetes, ALT, AST, UA, and HLP.

Among participants with MASLD, significant indirect effects were observed for DII through PHR (8.41, 95% CI: 2.06–19.74; *p* = 0.008) and platelets (9.19, 95% CI: 1.72–20.70; *p* = 0.018), yielding negative mediation proportions of −16.9% and −17.9%, respectively. Similarly, CDAI demonstrated significant indirect pathways via PHR (−1.09, 95% CI: −2.45 to −0.24; *p* = 0.008; proportion: 10.2%) and platelets (−1.18, 95% CI: −2.82 to −0.20; *p* = 0.018; proportion: −0.5%). Because the direct and indirect effects frequently operated in opposite directions, and given the absence of statistically significant total effects, these patterns indicate complex suppression effects (inconsistent mediation) rather than conventional partial mediation. Consequently, these findings suggest potential indirect associations rather than definitive mediating roles. In MetALD patients (Figure S3), no mediating effects were found between platelets and PHR for all indices (DII, CDAI, DAQS).

### 3.6 Subgroup Analyses and Sensitivity Analyses

Stratified Cox proportional hazards analyses weighted by age, sex, smoking status, BMI, FIB‐4, cancer, and diabetes were conducted to investigate associations between dietary indices (DII/CDAI/DAQS) and all‐cause mortality in MASLD/MetALD populations. Subgroup analyses revealed that these associations were significantly modified by age, sex, smoking status, BMI, FIB‐4, cancer, and diabetes, with distinct patterns observed across disease status (MASLD/MetALD). In the total population (Figure S4), when FIB‐4 was < 1.3, DII showed the strongest positive association with mortality (HR = 1.428), while CDAI (HR = 0.978) and DAQS (HR = 0.563) exhibited significant negative correlations. As FIB‐4 increased, the association strength weakened, with interaction *p‐*values of 0.007 (DII) and 0.003 (DAQS). In MASLD and MetALD patients (Figures S5 and S6), DII remained significantly associated (HR = 1.631) and CDAI showed a clear negative correlation (HR = 0.814) when FIB‐4 was < 1.3. However, when FIB‐4 exceeded 2.67, the association of DII became non‐significant, and the protective effects of CDAI and DAQS weakened.

Sensitivity analyses were conducted in the total population and among adults with MASLD and MetALD. After adjusting for different confounding factors (Model 1 adjusted for age, sex, etc.; Model 2 adjusted for FIB‐4, etc.), the associations between dietary indices and all‐cause mortality persisted. In the total population, DII was positively associated with mortality, while CDAI and DAQS showed negative associations. Similar trends were observed in adults with MASLD and MetALD, indicating robustness of the findings (see Table S3).

## 4 Discussion

This study systematically evaluated the relationship between dietary inflammatory potential (DII) and two dietary antioxidant indicators (CDAI, DAQS) with mortality risk through in‐depth analysis of the total population with SLD characteristics and SLD subgroups population. Findings indicate that associations between dietary inflammatory potential and antioxidant capacity with all‐cause mortality differ across the total population and MASLD/MetALD patients. Specifically, pro‐inflammatory diets (higher DII) significantly increased all‐cause mortality risk across all groups, with the strongest association observed in MetALD patients. Conversely, higher dietary antioxidant levels (CDAI) and higher antioxidant dietary quality (DAQS) exerted protective effects in the total SLD‐characterized population and MASLD population, though this benefit was not significant in MetALD patients. This study conducted a combined analysis to explore the effects of different dietary patterns and alcohol intake on all‐cause mortality risk in the total population with SLD characteristics. Individuals with the highest DII (T3) or lowest antioxidant markers (CDAI/DAQS) (T1) exhibited the highest mortality risk, even among light drinkers, and were designated as the control group. In contrast, individuals in the lowest DII percentile (T1) who consumed moderate amounts of alcohol exhibited the lowest overall mortality risk. Within the moderate‐drinking group, high dietary antioxidant levels (CDAI) and high antioxidant dietary quality (DAQS) demonstrated protective effects. This study offers a novel complementary perspective to current international guidelines: a high anti‐inflammatory diet combined with high‐quality antioxidant dietary patterns confers benefits in reducing all‐cause and cardiovascular mortality risks for individuals across the SLD spectrum. For those unable to achieve complete abstinence (MetALD population), enhancing dietary anti‐inflammatory capacity and adopting high‐quality antioxidant diets may represent critical life‐saving lifestyle interventions.

Inflammation and oxidative stress play pivotal roles in the pathogenesis of MASLD/MetALD (Gao et al. 2019; Clare et al. 2022). Research indicates dietary nutrients influence systemic inflammation and antioxidant status, with DII serving as a potential indicator of a diet's inflammatory potential (Shivappa et al. 2014), while CDAI/DAQS reflect comprehensive dietary antioxidant capacity and antioxidant quality, respectively. Our findings support existing evidence that pro‐inflammatory diets or diets low in antioxidant capacity increase mortality risk in MASLD populations. Specifically, our observations regarding the association between the DII and mortality risk in patients with MASLD are consistent with a recent study by Tao et al. (2025), underscoring the critical role of dietary inflammatory potential in shaping long‐term outcomes, consistent with our results and underscoring the key role of dietary inflammatory potential in shaping long‐term outcomes. Building upon this established consensus, our study further reveals that the detrimental effects of a pro‐inflammatory diet extend to the MetALD population, enhancing mortality risk in the context of moderate alcohol consumption. Moreover, by simultaneously evaluating multiple antioxidant dietary dimensions, we found that higher CDAI and DAQS independently exert protective effects on survival in both MASLD and MetALD. Regarding antioxidant diets, Zheng et al. (2024) reported that higher CDAI may be associated with lower FLI. Our findings extend these observations by demonstrating that both higher dietary antioxidant capacity and better antioxidant dietary quality are associated with reduced mortality risk in MASLD. In contrast, the associations of CDAI and DAQS with mortality did not reach statistical significance in MetALD. Biologically, we speculate that the effect of dietary quality on mortality risk may be modified by the SLD phenotype. In the within‐group analysis, the substantial heterogeneity in alcohol exposure among the MetALD cohort may have attenuated the independent effect of dietary factors (Yoo et al. 2020), thereby masking their independent protective effects in the separate analysis of the MetALD cohort. Methodologically, the limited number of mortality events observed in this specific subgroup might have resulted in insufficient statistical power to detect a true independent association. Given this limitation, focusing solely on the independent associations within each SLD subtype may not fully capture the combined influence of SLD subtype and dietary quality. We therefore conducted joint analyses to further examine mortality risk across different combinations of SLD subtype and each dietary index.

To further elucidate this complexity and explore the potential trends that might be obscured in isolated subgroup analyses, we conducted a joint analysis in the overall population with SLD characteristics. The results suggested that healthier dietary patterns were associated with lower mortality risk across different SLD subtypes. Several factors may contribute to this observation. First, in terms of baseline characteristics, patients with MetALD are generally younger and exhibit better dietary quality compared with those with MASLD; moreover, more favorable profiles are also observed among MetALD patients in composite inflammatory indices. These findings suggest that MetALD may possess a higher level of health management awareness (e.g., greater attention to balanced diet and routine health practices) (Barefoot et al. 2002), which may partially account for the observed differences in risk between the two groups. In addition, previous studies have suggested that moderate alcohol consumption may reduce cardiovascular mortality, potentially through improvements in endothelial function (Freeman et al. 2018), while alcohol exposure may simultaneously increase the risk of adverse liver‐related outcomes, including liver injury and hepatocellular carcinoma (Ochoa‐Allemant et al. 2025; Celsa et al. 2025). In the context of higher dietary quality, healthy dietary patterns, which characterized by high antioxidant and anti‐inflammatory components, may attenuate alcohol‐related hepatic damage by reducing systemic inflammation and improving metabolic status (Wang et al. 2023; Petermann‐Rocha et al. 2024). All in all, even in the context of moderate alcohol consumption, maintaining high antioxidant intake and low inflammatory intake appears to be associated with reduced mortality risk, suggesting that optimizing dietary habits could serve as a beneficial strategy for improving outcomes in individuals with MetALD. Nevertheless, these findings remain preliminary and require further validation in large population cohorts.

To explore potential mechanisms, we conducted exploratory mediation analyses of inflammatory markers. Among MASLD patients, dietary indices (DII and CDAI) showed indirect associations involving platelets and PHR. However, the observed negative mediation proportions point to inconsistent mediation or suppression effects. This suggests that while diet quality clearly influences inflammatory and blood markers, these markers do not act as simple, direct mediators. Instead, they are part of a more complex network of interacting biological pathways. In the MetALD subgroup, no statistically significant mediation effects were observed for the evaluated inflammatory markers. This may indicate that the relationship between dietary quality and mortality involves more complex pathways in the context of alcohol exposure. Alcohol‐related biological alterations (Smith et al. 2015), may influence circulating inflammatory and hematological markers, while other pathways such as gut microbiota regulation and hepatocellular protection may also contribute to the beneficial effects of dietary patterns (Gao et al. 2025; Abenavoli et al. 2017). Further studies are needed to clarify these potential mechanisms.

This study also has certain limitations. First, the findings from NHANES relied on self‐reported data, which may introduce recall bias. Second, a methodological limitation of this study involves the modified application of the EASL‐AASLD‐ALEH 2023 consensus criteria. Specifically, we omitted the Asian‐specific BMI cut‐off to maintain analytical consistency across the multi‐ethnic US cohort and excluded the OGTT criterion because its limited availability in NHANES subsamples would have severely compromised our statistical power. Third, the dietary nutrient intake used to calculate DII, CDAI, and DAQS was derived from the average of 24‐h dietary recalls, and it is not possible to accurately track the types and amounts of alcohol consumed by individuals, which may not fully represent the daily dietary intake of long‐term exposure. Fourth, due to the limitations of food components in NHANES, we did not include plant‐derived bioactive compounds with antioxidant properties in the calculation of dietary indices, which may result in certain biases in our results. Additionally, due to the absence of some mortality codes, the underestimation of the death events may increase the likelihood of Type II errors. This is particularly pertinent in the MetALD subgroup, where only 58 all‐cause mortality events were documented. Such limited statistical power underscores the need to interpret the non‐significant findings in this subgroup cautiously. Given the limited number of events in the MetALD subgroup, the potential interaction between alcohol consumption and dietary quality requires further investigation in larger populations. Moreover, given the limited absolute number of cardiovascular‐specific deaths, we were statistically precluded from performing a reliable mediation analysis for CVD mortality. Future studies involving larger and more diverse populations, together with advanced cardiovascular phenotyping approaches, are warranted to further elucidate whether favorable dietary patterns are associated with improved cardiovascular health beyond conventional metabolic outcomes in MASLD/MetALD (Mantovani et al. 2023; Sonaglioni et al. 2025).

Overall, these findings suggest dietary modifications benefit MASLD/MetALD patients and inform dietary recommendations for SLD patients. Current guidelines consistently emphasize that MASLD patients should limit alcohol intake and adopt anti‐inflammatory dietary strategies (following the Mediterranean diet) (Rinella, Neuschwander‐Tetri, et al. 2023). However, in practice, due to social factors or addictive tendencies, it is challenging for MetALD individuals (moderate drinkers with MASLD) to abstain completely. In managing the MetALD population, priority should be given to the anti‐inflammatory and antioxidant quality of the diet, in addition to conventional recommendations for alcohol restriction. By optimizing antioxidant dietary quality or reducing inflammatory dietary components, patients can achieve significant survival benefits even with moderate alcohol consumption. For instance, replacing refined grains with whole grains (e.g., oats, whole wheat), avoiding fried foods and processed red meat, and consuming fiber‐ and vitamin‐rich vegetables, fruits, and legumes can better mitigate oxidative stress (Chen et al. 2022). Dark‐colored fruits and vegetables, in particular, contain higher concentrations of antioxidants (Åhlberg 2021). Berries like blueberries and cranberries are rich in anthocyanins (plant pigments) and vitamin C (Kalt et al. 2020). Furthermore, our analysis indicates that improving dietary quality demonstrates stronger independent protective effects against all‐cause and cardiovascular mortality than alcohol restriction alone. Therefore, for patients with the SLD spectrum, it is recommended to enhance dietary antioxidant levels and quality in addition to limiting alcohol intake and adopting anti‐inflammatory dietary strategies. These findings may provide a foundation for developing future dietary guidelines for MASLD/MetALD, potentially yielding survival benefits for this patient population.

## 5 Conclusions

This study found that a pro‐inflammatory diet increases the risk of mortality in patients with SLD. Conversely, higher dietary antioxidant levels and quality (CDAI/DAQS) exert a protective effect on survival in the MASLD subgroup. Joint analysis reveals that a low‐inflammatory and high‐antioxidant diet can mitigate alcohol's adverse effects, so that even among MetALD, improving dietary anti‐inflammatory and antioxidant quality yields survival benefits. In conclusion, optimizing dietary anti‐inflammatory and antioxidant quality should serve as a key nutritional intervention strategy to improve the prognosis of patients across the SLD spectrum.

## Author Contributions

**Haixin Lyu:** conceptualization, writing – original draft, formal analysis. **Haiyan Chen:** writing – original draft, software, methodology. **Zegeng Zhan:** data curation, investigation. **Yangyang Qin:** data curation, investigation. **Lifang Mao:** methodology, software. **Meiyan Guo:** methodology, validation. **Yaxian Mo:** writing – review and editing, conceptualization. **Tianran Shen:** conceptualization, funding acquisition, writing – review and editing.

## Funding

This study was funded by the National Natural Science Foundation of China, grant number No. 82204028, Guangzhou Basic and Applied Basic Research Project, grant number 2025A04J5225, the 2024 High‐quality Development Research Project of Shenzhen Bao'an Public Hospital, grant number BAGZL2024188, and Guangdong Pharmaceutical University Special Project for “Cultivating Disciplinary Excellence and Enhancing Quality through Innovation”, grant number XKPYMS20260821.

## Ethics Statement

The studies involving humans were approved by the National Center for Health Statistics Review Board. The studies were conducted in accordance with the local legislation and institutional requirements.

## Consent

Informed consent was obtained from all subjects involved in the study.

## Conflicts of Interest

The authors declare no conflicts of interest.

## Supporting information


**Figure S1:** Survival curves using the Kaplan–Meier method for the patients with total, MASLD and MetALD.
**Figure S2:** Associations of DII, CDAI and DAQS with CVD mortality, evaluated using CPH regression models and RCS.
**Figure S3:** Analysis of the mediation by Platelet (A–C) and PHR (D–F) of the associations of dietary indices with all‐cause mortality among adults with MetALD.
**Figure S4:** Subgroup analyses of multivariable adjusted association of the dietary indices with all‐cause mortality among total adults.
**Figure S5:** Subgroup analyses of multivariable adjusted association of the dietary indices with all‐cause mortality among adults with MASLD.
**Figure S6:** Subgroup analyses of multivariable adjusted association of the dietary indices with all‐cause mortality among adults with MetALD.
**Table S1:** Association of dietary indices with CVD mortality in adults with total, MASLD and MetALD.
**Table S2:** Analysis of the mediation by inflammation‐related indicators of the associations of dietary indices with all‐cause mortality among adults with FLD‐MD, MASLD and MetALD.
**Table S3:** Sensitivity analyses of multi‐variable adjusted association of dietary indices with all‐cause mortality in adults with total, MASLD and MetALD.

## Data Availability

Publicly available datasets were analyzed in this study. The data can be found here: https://www.cdc.gov/nchs/nhanes/.
