# Supplementary material for: Differential Associations of Dietary Inflammatory Index and Dietary Antioxidant Indices With Mortality Risk From MASLD/MetALD: Results From a Large Population‐Based Study
Source: Food Sci Nutr. 2026 Oct 6;14(10):e72429. doi: 10.1002/fsn3.72429 (PMC13643365; doi:10.1002/fsn3.72429)
Supplement: Supplementary file 1 [file FSN3-14-e72429-s001.docx]

**Supplementary materials**

**Figures:**

Figure S1. Survival curves using the Kaplan-Meier method for the patients with Total,

MASLD and MetALD.

Figure S2. Associations of DII, CDAI and DAQS with CVD mortality, evaluated using

CPH regression models and RCS

Figure S3. Analysis of the mediation by Platelet(A-C) and PHR(D-F) of the associations of dietary indices with all-cause mortality among adults with MetALD.

Figure S4. Subgroup analyses of multivariable adjusted association of the dietary indices with all-cause mortality among total adults.

Figure S5. Subgroup analyses of multivariable adjusted association of the dietary indices with all-cause mortality among adults with MASLD.

Figure S6. Subgroup analyses of multivariable adjusted association of the dietary indices with all-cause mortality among adults with MetALD.

**Tables:**

Table S1. Association of dietary indices with CVD mortality in adults with Total, MASLD and MetALD

Table S2. Analysis of the mediation by inflammation-related indicators ofthe associations of dietary indices with all-cause mortality among adults with FLD-MD, MASLD and MetALD

Table S3. Sensitivity analyses of multi-variable adjusted association of dietary indices with all-cause mortality in adults with total, MASLD and MetALD

**Figure S1** Survival curves using the Kaplan-Meier method for the patients with Total, MASLD and MetALD. (A-C)Total, (D-F)adults with MASLD, (G-I)adults with MetALD. DII, Dietary Inflammatory Index; CDAI, composite dietary antioxidant index; DAQS, Dietary Antioxidant Quality Score.

**Figure S2** Associations of DII, CDAI and DAQS with CVD mortality, evaluated using CPH regression models and RCS after adjusting for age, sex, ethnicity, income, body mass index, smoking status, sitting time, total energy intake, dietary supplements use, self-reported cancer, diabetes, ALT, AST, UA and HLP. Central estimates are represented by solid blue lines, and red shaded areas indicate 95% confidence intervals. (A-C) total participants, (D-F) adults with MASLD, (G-I) adults with MetALD.

**Figure S3** Analysis of the mediation by Platelet(A-C) and PHR(D-F) of the associations of dietary indices with all-cause mortality among adults with MetALD. The 95% CI of these estimates was computed using the bootstrap method (1,000 samples). In all mediation analyses, adjustments were made for the following covariates: adjusted for age, sex, ethnicity, income, body mass index, smoking status, sitting time, total energy intake, dietary supplements use, self-reported cancer, diabetes, ALT, AST, UA and HLP.

**Figure S4** Subgroup analyses of multivariable adjusted association of the dietary indices with all-cause mortality among total adults.

**Figure S5** Subgroup analyses of multivariable adjusted association of the dietary indices with all-cause mortality among adults with MASLD.

**Figure S6** Subgroup analyses of multivariable adjusted association of the dietary indices with all-cause mortality among adults with MetALD.

**Table S1.** Association of dietary indices with CVD mortality in adults with Total, MASLD and MetALD

|  |  | T1 | T2 | | T3 | |  |
| --- | --- | --- | --- | --- | --- | --- | --- |
|  |  |  | HR | *P* | HR | *P* | *P-trend* |
| Total |  |  |  |  |  |  |  |
|  | DII |  |  |  |  |  |  |
|  | Model 1 | ref | 1.31 (0.63-2.76) | 0.468 | 1.57 (0.74-3.33) | 0.237 | 0.230 |
|  | Model 2 | ref | 1.40 (0.67-2.92) | 0.366 | 1.70 (0.81-3.56) | 0.159 | 0.150 |
|  | Model 3 | ref | 1.54 (0.70-3.36) | 0.278 | 1.84 (0.83-4.07) | 0.134 | 0.094 |
|  | CDAI |  |  |  |  |  |  |
|  | Model 1 | ref | 0.57 (0.27-1.21) | 0.277 | 0.53 (0.22-1.28) | 0.156 | 0.194 |
|  | Model 2 | ref | 0.55 (0.26-1.19) | 0.127 | 0.51 (0.22-1.18) | 0.113 | 0.152 |
|  | Model 3 | ref | 0.54 (0.21-1.36) | 0.186 | 0.40 (0.13-1.29) | 0.124 | 0.132 |
|  | DAQS |  |  |  |  |  |  |
|  | Model 1 | ref | 0.83 (0.58-1.19) | 0.315 | 0.55 (0.33-0.93) | 0.026 | 0.195 |
|  | Model 2 | ref | 0.82 (0.58-1.17) | 0.272 | 0.54 (0.32-0.90) | 0.019 | 0.016 |
|  | Model 3 | ref | 0.73 (0.50-1.08) | 0.117 | 0.42 (0.24-0.74) | 0.003 | 0.029 |
| MASLD |  |  |  |  |  |  |  |
|  | DII |  |  |  |  |  |  |
|  | Model 1 | ref | 0.90 (0.37-2.20) | 0.821 | 1.38 (0.59-3.27) | 0.456 | 0.493 |
|  | Model 2 | ref | 1.05 (0.42-2.62) | 0.921 | 1.65 (0.71-3.85) | 0.242 | 0.264 |
|  | Model 3 | ref | 1.25 (0.54-2.90) | 0.598 | 2.06 (0.81-5.24) | 0.127 | 0.136 |
|  | CDAI |  |  |  |  |  |  |
|  | Model 1 | ref | 0.68 (0.31-1.53) | 0.350 | 0.76 (0.30-1.91) | 0.549 | 0.606 |
|  | Model 2 | ref | 0.72 (0.32-1.63) | 0.425 | 0.74 (0.31-1.80) | 0.504 | 0.542 |
|  | Model 3 | ref | 0.73 (0.29-1.88) | 0.516 | 0.61 (0.21-1.81) | 0.368 | 0.374 |
|  | DAQS |  |  |  |  |  |  |
|  | Model 1 | ref | 0.64 (0.29-1.39) | 0.253 | 0.53 (0.15-1.92) | 0.332 | 0.284 |
|  | Model 2 | ref | 0.59 (0.27-1.31) | 0.589 | 0.41 (0.11-1.58) | 0.410 | 0.152 |
|  | Model 3 | ref | 0.59 (0.25-1.41) | 0.231 | 0.43 (0.11-1.68) | 0.220 | 0.184 |
| MetALD |  |  |  |  |  |  |  |
|  | DII |  |  |  |  |  |  |
|  | Model 1 | ref | 2.65 (0.70-10.10) | 0.151 | 1.93 (0.54-6.86) | 0.305 | 0.274 |
|  | Model 2 | ref | 2.99 (0.70-12.71) | 0.137 | 1.98 (1.41-9.65) | 0.394 | 0.359 |
|  | Model 3 | ref | 4.55 (0.81-25.58) | 0.084 | 2.72 (0.43-17.23) | 0.284 | 0.266 |
|  | CDAI |  |  |  |  |  |  |
|  | Model 1 | ref | 0.34 (0.05-2.28) | 0.263 | 0.29 (0.05-1.71) | 0.170 | 0.210 |
|  | Model 2 | ref | 0.30 (0.02-3.47) | 0.344 | 0.26 (0.04-1.38) | 0.114 | 0.171 |
|  | Model 3 | ref | 0.13 (0.01-2.24) | 0.134 | 0.10 (0.01-2.19) | 0.094 | 0.226 |
|  | DAQS |  |  |  |  |  |  |
|  | Model 1 | ref | 2.51 (0.47-13.47) | 0.278 | 0.37 (0.08-1.62) | 0.182 | 0.278 |
|  | Model 2 | ref | 2.69 (0.52-13.88) | 0.234 | 0.37 (0.08-1.74) | 0.205 | 0.358 |
|  | Model 3 | ref | 2.14 (0.32-14.08) | 0.427 | 0.21 (0.05-0.97) | 0.046 | 0.149 |

Model 1, adjusted for age, sex, ethnicity and income. Model 2, model 1 + body mass index, smoking status and sitting time. Model 3, model 2 + total energy intake, dietary supplements use, cancer, diabetes, ALT, AST, UA and HLP.

**Table S2** Analysis of the mediation by inflammation-related indicators of the associations of dietary indices with all-cause mortality among adults with total, MASLD and MetALD

|  |  | **Mediation effect (95% CI), *P* value** | | | |
| --- | --- | --- | --- | --- | --- |
|  |  | Total effect | Indirect effect | Direct effect | Mediation |
| **Total** |  |  |  |  |  |
| **DII** |  |  |  |  |  |
|  | UHR | -47.27(-114.90, 7.31) 0.094 | 1.95(-0.94, 5.80) 0.186 | -49.22(-115.59, 5.80) 0.086 | -3.7% |
|  | NLR | -48.17(-111.73, 2.95) 0.070 | -0.84(-3.44, 1.53) 0.470 | -47.32(-110.78, 3.10) 0.070 | 1.6% |
|  | PLR | -44.67(-108.86, 7.96) 0.110 | -1.74(-4.90, 0.03) 0.060 | -42.93(-106.22, 9.27) 0.120 | 3.4% |
|  | SII | -48.42(-112.39, 4.75) 0.078 | **-2.04(-5.15, -0.08) 0.036** | -46.37(-109.43, 6.01) 0.094 | 3.9% |
|  | PHR | -37.83(-97.57, 14.70) 0.162 | **7.96(2.68, 15.98) <0.001** | -45.79(-111.54, 7.11) 0.094 | -18.4% |
|  | Neutrophil | -48.71(-115.59, 5.00) 0.078 | -2.07(-5.94, 0.22) 0.098 | -46.64(-112.60, 6.28) 0.092 | 3.7% |
|  | Lymphocyte | -43.30(-108.00, 9.11) 0.120 | 0.10(-0.90, 1.18) 0.970 | -43.4(-108.00, 9.23) 0.120 | 0.1% |
|  | Platelet | -36.99(-97.57, 15.09) 0.174 | **9.40(2.91, 18.42) <0.001** | -46.38(-109.99, 6.27) 0.086 | -21.4% |
| **CDAI** |  |  |  |  |  |
|  | UHR | 5.47(-1.12, 13.10) 0.100 | -0.09(-0.45, 0.22) 0.550 | 5.56(-0.95, 13.18) 0.100 | -1.3% |
|  | NLR | 4.61(-1.22, 11.50) 0.130 | 0.15(-0.07, 0.42) 0.200 | 4.47(-1.17, 10.77) 0.140 | 2.7% |
|  | PLR | 4.85(-1.49, 11.87) 0.146 | **0.23(0.01, 0.57) 0.038** | 4.62(-1.67, 11.56) 0.166 | 3.8% |
|  | SII | 5.03(-1.22, 11.84) 0.120 | **0.20(-0.001, 0.48) 0.056** | 4.83(-1.19, 11.64) 0.134 | 3.3% |
|  | PHR | 4.88(-1.37, 11.68) 0.146 | **-0.64(-1.47, -0.13) 0.012** | 5.52(-0.85, 12.46) 0.096 | -11.1% |
|  | Neutrophil | 5.69(-0.71, 13.15) 0.082 | 0.12(-0.12, 0.44) 0.338 | 5.56(-0.79, 13.07) 0.098 | 1.9% |
|  | Lymphocyte | 5.00(-1.25, 12.30) 0.140 | -0.03(-0.16, 0.05) 0.510 | 5.03(-1.29, 12.27) 0.140 | -0.4% |
|  | Platelet | 5.09(-1.10, 11.98) 0.136 | **-0.74(-1.72,-0.10) 0.024** | **5.83(-0.62, 12.76) 0.080** | -12.4% |
| **DAQs** |  |  |  |  |  |
|  | UHR | 54.20(-1.84, 109.01) 0.066 | -1.42(-5.33, 1.64) 0.338 | 55.62(-0.38, 110.76) 0.054 | -2.1% |
|  | NLR | 49.22(-2.94, 99.35) 0.074 | 0.20(-2.16, 2.64) 0.876 | 49.02 (-3.38, 99.34) 0.072 | 0.4% |
|  | PLR | 48.94(-8.19, 101.93) 0.090 | 0.40(-1.56, 2.68) 0.652 | 48.54 (-9.12, 101.56) 0.088 | 0.6% |
|  | SII | 51.07(-3.94, 103.19) 0.074 | 1.37(-0.56, 4.00) 0.176 | 49.70(-5.69, 101.47) 0.080 | 2.3% |
|  | PHR | 51.11(-4.47, 101.89) 0.066 | **-5.50(-13.56, -0.40) 0.034** | **56.61(2.96, 107.76) 0.046** | -9.4% |
|  | Neutrophil | 55.07(0.79, 108.28) 0.050 | 1.88(-0.44, 5.27) 0.134 | 53.20(-1.39, 105.67) 0.058 | 3.0% |
|  | Lymphocyte | 50.65(-5.55, 103.09) 0.080 | 0.37(-0.43, 1.68) 0.430 | 50.28(-6.27, 102.69) 0.080 | 0.5% |
|  | Platelet | 51.01(-5.04, 102.29) 0.074 | -6.01(-15.49, 0.25) 0.066 | **57.02(2.27, 109.10) 0.046** | -0.1% |
| **MASLD** |  |  |  |  |  |
| **DII** |  |  |  |  |  |
|  | UHR | -19.00(-87.50, 39.85) 0.560 | 0.17(-3.75, 3.72) 0.920 | -19.10(-85.20, 40.84) 0.560 | -0.003% |
|  | NLR | -24.64(-89.57, 30.47) 0.390 | 0.68(-1.97, 3.41) 0.560 | -25.32(-89.33, 29.99) 0.390 | -1.4% |
|  | PLR | -17.88(-84.43, 39.36) 0.850 | -0.51(-3.09, 1.02) 0.570 | -17.37(-83.58, 39.94) 0.590 | 0.4% |
|  | SII | -21.71(-88.18, 35.36) 0.500 | -0.50(-3.22, 1.44) 0.600 | -21.20(-87.30, 35.84) 0.500 | 0.8% |
|  | PHR | -13.49(-73.61, 43.44) 0.700 | **8.41(2.06, 19.74) 0.008** | -21.89(-86.07, 35.29) 0.486 | -16.9% |
|  | Neutrophil | -21.52(-88.80, 35.88) 0.490 | -1.10(-4.86, 1.16) 0.390 | -20.42(-86.29, 37.20) 0.520 | 1.7% |
|  | Lymphocyte | -18.10(-84.00, 39.25) 0.230 | 0.03(-83.70, 38.95) 0.570 | -18.10(-83.70, 38.95) 0.570 | -0.02% |
|  | Platelet | -13.32(-74.84, 43.79) 0.700 | **9.19(1.72, 20.70) 0.018** | -0.18(-4.11, 5.38) 0.706 | -17.9% |
| **CDAI** |  |  |  |  |  |
|  | UHR | 0.26(-7.66, 8.70) 0.980 | 0.12(-0.31, 0.63) 0.580 | 0.14(-7.75, 8.47) 1.000 | 0.2% |
|  | NLR | 0.17(-7.17, 7.64) 0.980 | 0.06(-0.24, 0.39) 0.670 | 0.10(-7.30, 7.68) 0.990 | 0.1% |
|  | PLR | -0.23(-8.14, 7.93) 0.950 | 0.20(-0.08, 0.65) 0.190 | -0.42(-8.35, 7.80) 0.910 | -0.5% |
|  | SII | 0.08(-7.73, 8.08) 1.000 | 0.12(-0.11, 0.43) 0.280 | -0.04(-7.87, 7.98) 0.970 | -0.007% |
|  | PHR | -0.18(-7.89, 7.62) 0.950 | **-1.09(-2.45, -0.24) 0.008** | 0.91(-6.56, 8.76) 0.802 | 10.2% |
|  | Neutrophil | 0.65(-7.19, 8.92) 0.880 | 0.06(-0.24, 0.42) 0.690 | 0.59(-7.39, 9.05) 0.890 | 0.3% |
|  | Lymphocyte | 0.01(-7.99, 8.25) 0.980 | -0.05(-0.27, 0.08) 0.550 | 0.06(-8.04, 8.29) 0.100 | -0.06% |
|  | Platelet | 0.08(-7.62, 7.83) 0.990 | **-1.18(-2.82, -0.20) 0.018** | 1.26(-6.26, 9.19) 0.750 | -0.5% |
| **DAQs** |  |  |  |  |  |
|  | UHR | 28.39(-53.29, 100.56) 0.420 | 0.22(-4.38, 4.66) 0.910 | 28.17(-53.97, 100.67) 0.420 | 0.4% |
|  | NLR | 26.88(-52.30, 94.10) 0.410 | -1.02(-4.60, 1.95) 0.500 | 27.90(-49.11, 96.61) 0.400 | -1.6% |
|  | PLR | 20.70(-66.90, 93.86) 0.540 | -0.30(-3.21, 1.81) 0.830 | 21.00(-66.20, 93.32) 0.530 | -0.08% |
|  | SII | 24.46(-58.84, 95.69) 0.470 | 1.24(-0.82, 4.29) 0.920 | 24.34(-57.69, 94.75) 0.470 | 0.1% |
|  | PHR | 26.36(-54.80, 94.62) 0.440 | -5.76(-16.40, 1.81) 0.120 | 32.12(-49.42, 102.86) 0.350 | -8.4% |
|  | Neutrophil | 29.84(-51.64, 99.24) 0.380 | 1.06(-1.99, 4.73) 0.480 | 28.78(-52.84, 99.03) 0.400 | 1.3% |
|  | Lymphocyte | 25.15(-58.50, 95.67) 0.460 | 0.54(-0.81, 2.59) 0.460 | 24.61(-58.83, 95.62) 0.470 | 0.5% |
|  | Platelet | 26.68(-56.19, 97.09) 0.440 | -6.01(-19.20, 3.44) 0.200 | 32.69(-48.59, 103.03) 0.340 | -8.4% |
| **Met-ALD** |  |  |  |  |  |
| **DII** |  |  |  |  |  |
|  | UHR | -161.67(-467.17, 43.44) 0.130 | -8.26(-38.07, 7.05) 0.360 | -153.42(-467.73, 46.66) 0.160 | 3.9% |
|  | NLR | -150.51(-443.86, 39.26) 0.140 | -3.86(-16.31, 1.77) 0.260 | -146.65(-447.17, 44.34) 0.150 | 1.9% |
|  | PLR | -159.00(-482.00, 37.37) 0.130 | -0.88(-20.50, 16.10) 0.940 | -158.00(-473.00, 41.49) 0.130 | 0.3% |
|  | SII | -154.18(-473.06, 38.37) 0.140 | -5.12(-22.98, 6.96) 0.420 | -149.06(-461.77, 42.97) 0.150 | 2.4% |
|  | PHR | -165.31(-487.31, 37.90) 0.110 | 10.45(-7.17, 38.11) 0.212 | -175.76(-510.49, 27.90) 0.094 | -5.2% |
|  | Neutrophil | -140.84(-422.33, 34.02) 0.130 | -4.62(-15.92, 2.14) 0.200 | -136.21(-413.03, 39.75) 0.140 | 2.9% |
|  | Lymphocyte | -137.19(-405.11, 41.18) 0.150 | -3.42(-15.62,4.73) 0.390 | -133.77(-407.79, 43.20) 0.150 | 2.0% |
|  | Platelet | -162.27(-470.79, 35.05) 0.114 | 10.06(-14.29,40.71) 0.382 | -172.33(-487.87, 27.02) 0.094 | -5.4% |
| **CDAI** |  |  |  |  |  |
|  | UHR | 21.19(-1.61, 58.74) 0.084 | 0.75(-0.79, 3.24) 0.364 | 20.44(-2.59, 59.21) 0.094 | 2.7% |
|  | NLR | 19.55(-1.95, 56.23) 0.084 | 0.30(-0.23, 1.28) 0.284 | 19.25(-2.10, 55.82) 0.090 | 1.1% |
|  | PLR | 21.02(-1.45, 60.01) 0.076 | 0.06(-0.94, 1.09) 0.868 | 20.97(-1.31, 59.16) 0.078 | 0.2% |
|  | SII | 20.34(-1.58, 57.44) 0.084 | 0.28(-0.44, 1.35) 0.450 | 20.06(-2.17, 57.24) 0.090 | 1.0% |
|  | PHR | 21.69(-0.78, 58.35) 0.070 | -0.16(-1.22, 0.74) 0.698 | 21.85(-0.61, 58.58) 0.068 | -0.4% |
|  | Neutrophil | 17.73(-1.65, 50.02) 0.088 | 0.28(-0.38, 1.14) 0.366 | 17.45(-2.02, 49.75) 0.094 | 1.4% |
|  | Lymphocyte | 18.24(-1.38, 51.33) 0.082 | -0.30(-1.34, 0.44) 0.422 | 18.54(-1.18, 50.71) 0.076 | -1.5% |
|  | Platelet | 21.47(-0.82, 59.21) 0.076 | -0.30(-1.59, 0.45) 0.454 | 21.77(-0.82, 60.06) 0.068 | -1.0% |
| **DAQs** |  |  |  |  |  |
|  | UHR | 201.22(19.52, 458.41) 0.034 | 4.53(-5.62, 21.51) 0.446 | 196.69(-16.15, 459.72) 0.036 | 1.5% |
|  | NLR | 188.59(18.46, 430.26) 0.036 | -1.37(-9.42, 3.62) 0.638 | 189.97(18.60, 435.72) 0.034 | -3.9% |
|  | PLR | 194.16(18.31, 445.97) 0.034 | 0.38(-6.99, 7.84) 0.856 | 193.78(16.51, 443.93) 0.034 | 0.1% |
|  | SII | 191.85(16.86, 441.36) 0.034 | 1.95(-3.28, 10.35) 0.474 | 189.90(15.35, 436.48) 0.034 | 0.7% |
|  | PHR | 199.41(23.13, 449.72) 0.032 | -8.99(-32.51, 4.18) 0.198 | 208.41(33.94, 467.92) 0.030 | -3.8% |
|  | Neutrophil | 175.19(20.30, 394.39) 0.032 | 0.89(-6.14, 8.53) 0.774 | 174.30(16.42, 394.89) 0.032 | 0.5% |
|  | Lymphocyte | 167.34(3.35, 396.61) 0.046 | 5.82(-0.98, 17.47) 0.096 | 161.52(-3.00, 382.99) 0.056 | 3.1% |
|  | Platelet | 196.63(25.03, 443.23) 0.030 | -7.80(-32.74, 7.85) 0.340 | **204.43(28.38, 451.78) 0.030** | -3.5% |

Adjust for: age, sex, ethnicity, income, body mass index, smoking status, sitting time, total energy intake, dietary supplements use, self-reported cancer, diabetes, ALT, AST, UA and HLP.

**Table S3** Sensitivity analyses of multi-variable adjusted association of dietary indices with all-cause mortality in adults with total, MASLD and MetALD

|  |  | T1 | T2 | | T3 | |  |
| --- | --- | --- | --- | --- | --- | --- | --- |
|  |  |  | HR | *P* | HR | *P* | *P-trend* |
| **Total** |  |  |  |  |  |  |  |
|  | **DII** |  |  |  |  |  |  |
|  | Model 1 | ref | 1.67 (1.04-2.69) | 0.036 | 1.99 (1.24-2.69) | 0.005 | 0.004 |
|  | Model 2 | ref | 1.64 (1.04-2.57) | 0.034 | 2.04 (1.30-3.22) | 0.002 | 0.002 |
|  | **CDAI** |  |  |  |  |  |  |
|  | Model 1 | ref | 0.80 (0.56-1.15) | 0.225 | 0.47 (0.28-0.78) | 0.004 | 0.003 |
|  | Model 2 | ref | 0.74 (0.49-1.16) | 0.149 | 0.45 (0.27-0.74) | 0.002 | 0.002 |
|  | **DAQS** |  |  |  |  |  |  |
|  | Model 1 | ref | 0.76 (0.53-1.11) | 0.153 | 0.45 (0.26-0.78) | 0.005 | 0.005 |
|  | Model 2 | ref | 0.72 (0.49-1.06) | 0.099 | 0.48 (0.28-0.82) | 0.008 | 0.007 |
| **MASLD** |  |  |  |  |  |  |  |
|  | **DII** |  |  |  |  |  |  |
|  | Model 1 | ref | 1.32 (0.78-2.25) | 0.297 | 1.89 (1.04-3.43) | 0.036 | 0.035 |
|  | Model 2 | ref | 1.30 (0.78-2.15 | 0.309 | 1.91 (1.12-3.26) | 0.018 | 0.018 |
|  | **CDAI** |  |  |  |  |  |  |
|  | Model 1 | ref | 0.85 (0.53-1.31) | 0.454 | 0.52 (0.30-0.91) | 0.023 | 0.020 |
|  | Model 2 | ref | 0.80 (0.50-1.29) | 0.361 | 0.49 (0.28-0.86) | 0.013 | 0.011 |
|  | **DAQS** |  |  |  |  |  |  |
|  | Model 1 | ref | 0.63 (0.41-0.99) | 0.043 | 0.38 (0.19-0.78) | 0.008 | 0.007 |
|  | Model 2 | ref | 0.59 (0.36-0.96) | 0.036 | 0.43 (0.22-0.84) | 0.013 | 0.011 |
| **MetALD** |  |  |  |  |  |  |  |
|  | **DII** |  |  |  |  |  |  |
|  | Model 1 | ref | 4.97 (1.90-13.01) | 0.001 | 3.92 (1.47-10.43) | 0.007 | 0.004 |
|  | Model 2 | ref | 5.42 (2.10-14.03) | <0.001 | 4.43 (1.56-12.58) | 0.006 | 0.004 |
|  | **CDAI** |  |  |  |  |  |  |
|  | Model 1 | ref | 0.40 (0.11-1.54) | 0.181 | 0.23 (0.05-1.15) | 0.074 | 0.077 |
|  | Model 2 | ref | 0.51 (0.12-2.27) | 0.375 | 0.28 (0.05-1.60) | 0.151 | 0.123 |
|  | **DAQS** |  |  |  |  |  |  |
|  | Model 1 | ref | 1.41 (0.60-3.28) | 0.428 | 0.60 (0.25-1.40) | 0.232 | 0.140 |
|  | Model 2 | ref | 1.69 (0.67-4.25) | 0.262 | 0.68 (0.29-1.62) | 0.378 | 0.232 |

Model1: adjusted for age, sex, ethnicity, income, body mass index, smoking status, sitting time, total energy intake, dietary supplements use, self-reported cancer, diabetes, ALT, AST, UA, HDL-c, hypertension.

Model2: adjusted for age, sex, ethnicity, income, body mass index, smoking status, sitting time, total energy intake, dietary supplements use, self-reported cancer, diabetes, UA, HDL-c, and FIB-4.
